# Developing and applying synergistic multilevel implementation strategies to promote reach of an evidence-based parenting intervention in primary care

**DOI:** 10.1177/26334895221091219

**Published:** 2022-04-13

**Authors:** Samantha Schilling, Luisa Bigal, Byron J. Powell

**Affiliations:** 1 Department of Pediatrics, Division of General Pediatrics and Adolescent Medicine, 6797 University of North Carolina at Chapel Hill School of Medicine, Chapel Hill, NC, USA; 2 Center for Mental Health Services Research, Brown School and School of Medicine, Washington University in St. Louis, St. Louis, MO, USA

**Keywords:** parenting intervention, pediatric primary care, implementation, multilevel implementation strategies, implementation barriers

## Abstract

**Background:** This practical implementation report describes a primary care-based group parenting intervention—Child–Adult Relationship Enhancement in Primary Care (PriCARE)—and the approach taken to understand and strengthen the referral process for PriCARE within a pediatric primary care clinic through the deployment of synergistic implementation strategies to promote physician referrals. PriCARE has evidence of effectiveness for reducing child behavior problems, harsh and permissive parenting, and parent stress from three randomized controlled trials (RCTs). The integration of evidence-based parenting interventions into pediatric primary care is a promising means for widespread dissemination. Yet, even when integrated into this setting, the true reach will depend on parents knowing about and attending the intervention. A key factor in this process is the endorsement of and referral to the intervention by the child's pediatrician. Therefore, identifying strategies to improve physician referrals to parenting interventions embedded in primary care is worthy of investigation. **Method:** Through lessons learned from the RCTs and key informant interviews with stakeholders, we identified barriers and facilitators to physician referrals of eligible parent–child dyads to PriCARE. Based on this data, we selected and implemented five strategies to increase the PriCARE referral rate. We outline the selection process, the postulated synergistic interactions, and the results of these efforts. **Conclusions:** The following five discrete strategies were implemented: physician reminders, direct advertising to patients, incentives/public recognition, interpersonal patient narratives, and audit and feedback. These discrete strategies were synergistically combined to create a multifaceted approach to improve physician referrals. Following implementation, referrals increased from 13% to 55%. Continued development, application, and evaluation of implementation strategies to promote the uptake of evidence-based parenting interventions into general use in the primary care setting are discussed.

**Plain Language Summary:**

There is strong evidence that parenting interventions are effective at improving child behavioral health outcomes when delivered in coordination with pediatric primary care. However, there is a lack of focus on the implementation, including the screening and referral process, of parenting interventions in the primary care setting. This is contributing to the delay in the scale-up of parenting interventions and to achieving public health impact. To address this gap, we identified barriers and facilitators to physician screening and referrals to a primary care-based parenting intervention, and selected and piloted five synergistic strategies to improve this critical process. This effort successfully increased physician referrals of eligible patients to the intervention from 13% to 55%. This demonstration project may help advance the implementation of evidence-based interventions by providing an example of how to develop and execute multilevel strategies to improve intervention referrals in a local context.

Early behavioral problems affect 20% of all children under the age of 5 years in the United States and disproportionally impact children living in poverty (Egger & Angold, 2006). Such problems are associated with impairments in multiple domains, including family, academic, and social functioning, which often continue into adulthood (Campbell et al., 2006; Sayal et al., 2015). Thus, addressing behavior problems early has important downstream implications: children with behavior problems are disadvantaged in language, motor, social, and school readiness skills and are at increased risk of poor long-term academic and mental health outcomes (Montes et al., 2012; Wagner & Cameto, 2004; Weitzman & Wegner, 2015).

Parenting programs have been developed as short-term interventions that aim to improve parent–child relationships and address early child behavior problems (Furlong et al., 2013). Grounded in attachment and social learning theories, ample research provides evidence of the effectiveness of parenting programs in reducing challenging behavior (Barlow et al., 2005; Furlong et al., 2013) and improving educational (Hallam et al., 2006) and mental health outcomes (Barlow et al., 2005) in children, as well as reducing parent stress (Reyno & McGrath, 2006). In the long term, parenting programs also reduce substance use and delinquency (Sandler et al., 2011).

Indeed, the positive effects of these programs indicate promise for broad public health impact on children's well-being. Yet, their full potential has not been realized because their reach has been limited. One possible avenue to improve the dissemination of parenting programs is integration into pediatric primary care (Wakschlag et al., 2019). Within a span of 6 months, more than 75% of all U.S. children aged 0–18 years have had contact with their pediatrician making pediatric primary care a compelling service context for reaching families (*National Center for Health Statictics*, 201*8*; Smith et al., 2020). Providing parenting programs that target improving child behavioral problems is a particularly good fit within this setting because children are nearly always accompanied by their parents to well-child visits where recommendations regarding child behavior are often sought by parents (Leslie et al., 2016). There is also precedent for providing interventions inclusive of parents in pediatric primary care. For example, postpartum depression screening is now covered under children's health insurance when delivered at pediatric well-child visits (Liberto, 2012). This shift to supporting caregiver well-being provides an opening for the delivery of parenting interventions in pediatric primary care.

Although pediatric primary care is a promising venue for parenting interventions, barriers to implementation and dissemination abound (Baumann et al., 2015; Damschroder et al., 2009; Fixsen et al., 2009; Greenhalgh et al., 2004) and scholarship in this area is critical to achieving widespread improvements in children's emotional and behavioral health. For example, even when parenting interventions are delivered in pediatric primary care, there must be a procedure in place for eligible parents to be informed about the opportunity. Pediatric clinics with co-located parenting interventions often rely on endorsement and referral by the pediatrician during eligible patient encounters for this purpose (Kolko et al., 2014; Wildman & Langkamp, 2012). Pediatricians are a logical referral source since parents seek out and desire discussions with their child's pediatrician about child behavior, discipline, and parenting (Harwood et al., 2009; Miller & Sambell, 2003) and these topics are well aligned with recommended well-child visit content (Hagan et al., 2017). In addition, leveraging the relationship with the primary care provider in the referral process may help overcome barriers related to the perceived stigma of receiving parenting support and distrust of a new provider (Dempster et al., 2013; Leslie et al., 2016). However, regardless of who makes the referrals, in order for parenting interventions to be successful in pediatric primary care, there must be a strong screening and referral process: if children and families are not screened for eligibility and referred to the intervention, the co-located intervention will not be sustainable because of too few participants. Most research on parenting interventions targets the effectiveness of the intervention itself, with relatively little work to date focused on understanding the screening and referral processes.

In this practical implementation report, we describe a primary care-based parenting program—Child–Adult Relationship Enhancement in Primary Care (PriCARE)—and the approach taken to improve the referral rate for PriCARE within a pediatric primary care clinic through the deployment of implementation strategies that were intended to work synergistically to promote physician referrals. Weiner et al. (2012) suggest that when multiple strategies are combined synergistically, targeting implementation determinants at multiple levels of the social ecology, they mutually reinforce each other, thereby producing larger and longer-lasting effects than strategies that target determinates at only one level.

## Child–Adult Relationship Enhancement in Primary Care

PriCARE is an evidence-based intervention that has demonstrated decreases in child behavior problems, harsh and permissive parenting, and parent stress (Gurwitch et al., 2016; Schilling et al., 2017, 2020; Wood et al., 2021). *Criando Niños con Cariño* (Raising Children with Care) is a cultural adaptation of PriCARE for Spanish-speaking, Hispanic parents (Schilling et al., under review). PriCARE/*Cariño* is a manualized skill-based program delivered in the primary care setting by two licensed clinical social workers (LCSWs) during 6 weekly 90-minute sessions to groups of 6–10 parents. The LCSWs who deliver *Cariño* is bilingual. The curriculum aligns with adult learning theory and relies extensively on brainstorm activities, role play, and live coaching. Sessions 1–4 teach parenting skills focused on giving attention to children's positive, prosocial behaviors, while ignoring minor misbehaviors (strategic ignoring). Mastery of the 3 P skills (Praise, Paraphrase, and Point-out-Behavior) helps parents learn how to promote positive behaviors in their children. Sessions 5–6 teach techniques for giving children effective commands to set age-appropriate limits. The importance of play in supporting a child's development and establishing a strong foundation for the relationship between the child and parent is emphasized. Parents are encouraged to practice the PriCARE skills at home during brief (3–5 min) one-on-one play sessions with their child daily. PriCARE includes a trauma and stress education component that contextualizes the use of these skills with the types of behaviors and problems exhibited by many children living with psychosocial adversity and chronic familial stress. In response to parent feedback requesting text-based communication and reinforcement of key messages/skills between in-person sessions, a text messaging system was created that provides tips, reminders, and encouragement.

## Approach

### The Problem

In 2017, PriCARE was integrated into a pediatric clinic, with initial grant funding to support implementation. In 2019, a second grant was awarded to support the cultural adaptation of PriCARE for Spanish-speaking Hispanic families. Upon completion of the cultural adaptation in early 2020, the adapted program *Cariño* was also provided in the clinic. The primary referral source for PriCARE/*Cariño* is physicians who are asked to invite all eligible parent–child dyads (2–6-year-old patients with English or Spanish-speaking parents) to participate during well-child visits. Because of the necessity to maintain physical distancing during the COVID-19 pandemic, PriCARE/*Cariño* was transitioned to a virtual platform in April 2020.

After depletion of grant funding, the clinic elected to continue to offer PriCARE/*Cariño* to clinic families. However, the program did not receive a sufficient number of physician referrals to maintain ongoing intervention groups in spite of ample eligible child visits at the clinic. Only about 50% of parents who are referred enroll, and among those enrolled only about 80% attend at least one session. To continuously provide one PriCARE group and one *Cariño* group (each attended by 6–10 parents), about 10 referrals per week are required. With additional referrals, there is capacity to run multiple groups simultaneously. At the time this project was initiated, there were 0–2 PriCARE/*Cariño* referrals per week.

According to the policy activities that constitute research at our institution, this work met the criteria for operational improvement activities exempt from institutional review. Specifically, this was considered a quality improvement project focused on improving a local setting and therefore did not meet the criteria for IRB review.

### The Setting

The pediatric clinic is associated with an academic institution in a suburban town situated in a southeastern state. Based on data from the electronic medical record (EMR), the clinic cared for about 12,000 patients in 2020, including 1,385 unique 2–6-year-old children. Of all patients, 50% are female, 72% are insured by Medicaid or CHIP, 32% are Hispanic, 34% are non-Hispanic Black, 27% are non-Hispanic White, and 22% identify their preferred language as Spanish. During the time of this project, 52 physicians provided direct patient care at the clinic, three of whom spoke Spanish and English. The 52 physicians are divided into five teams to improve continuity of care such that on any given day, each team is represented in the clinic. The clinic had a full-time in-person Spanish interpreter in addition to the availability of virtual interpreters. One of the authors of this report is a physician at the clinic and supervises the PriCARE program.

### Identifying Barriers and Facilitators

We started by interviewing stakeholders to identify barriers and facilitators to physician referrals of eligible parent–child dyads to PriCARE. We invited the medical director, the associate medical director, and the 52 physicians providing direct patient care to participate in brief open-ended interviews. A total of 10 of 52 physicians, in addition to the medical director and associate medical director, replied to the invitation and were included for a total of 12 stakeholder interviews. During these stakeholder interviews, we asked the physician to describe their experiences with the current PriCARE referral process. Then we asked what was working well about the current process, and what made it challenging. We ended by requesting specific suggestions for improvement. The objective of these interviews was not to reach thematic saturation but rather was to help inform the selection of implementation strategies in a pragmatic manner. After interviewing clinic leadership (N = 2) and 10 physicians providing direct patient care, multiple potential targets for implementation strategies were identified (Table 1). In this practical implementation report, we present the selection and deployment of synergistic implementation strategies (Weiner et al., 2012) to increase the PriCARE referral rate, and the results of these efforts.

**Table 1.** Barriers and facilitators to Child–Adult Relationship Enhancement in Primary Care (PriCARE) referrals.

Barrier	Illustrative Quote
Complexity	The referral process is too complicated. I have to remember to ask the patient during the visit and document it in the note then send a message to the coordinator after and I have to remember her name.
Forgetting	I forget about it during the appointment and then remember when I’m writing my note and I fill out the PriCARE prompt.
Not a priority	There are a million things to do during well visits. Sometimes PriCARE doesn't make the list.
Knowledge/Time	Sometimes families have questions and I’m not sure if I can answer them or if it will take too much time.
Lack of recognition	There are so many initiatives and everyone has their pet project. Sometimes I think we fail to recognize and acknowledge how hard everyone is working to cover everything in 20 min.
**Facilitator**	**Illustrative Quote**
Patient need	So many families ask about behavior problems, I love having something at the clinic and free to offer them.
Reminder	I pre-chart and I make a note to ask about PriCARE.
Patient endorsement	I have had a few families who really rave about it. They say it was a gamechanger for them.

### Discrete Strategy Selection

We started with the discrete implementation strategies from the compilation identified in the Expert Recommendations for Implementation Change (ERIC) project to address the barriers that emerged during stakeholder interviews (Powell et al., 2015). We then incorporated theory, published and practice-based evidence, and stakeholder input to select and tailor implementation strategies that best addressed our determinants. Ultimately, five strategies targeting physician referrals were selected for implementation.

#### Strategy 1 (Physician Reminders)

**Figure 1.**
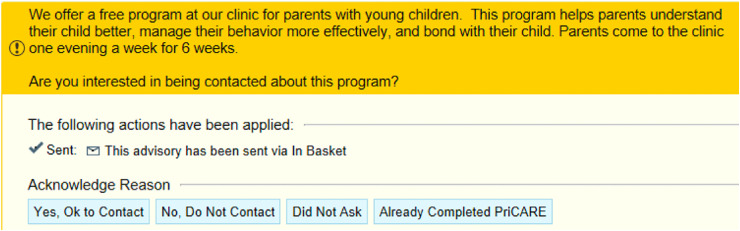
Electronic medical record (EMR) alert for Child–Adult Relationship Enhancement in Primary Care (PriCARE).

Stakeholder interviews revealed problems with the complexity of the current referral process to PriCARE and of the compatibility of fitting referrals into the current workflow during patient encounters. A strategy to remind physicians to refer patients to PriCARE could address these barriers. Rational decision-making theories assume an analytical model in which professionals consider and balance advantages and disadvantages to provide optimal care (Wensing & Grol, 2020). Decision support such as point-of-care alerts in the EMR could play an important role in this process (Shojania et al., 2021). Using the PriCARE referral criteria, an EMR alert was designed and embedded in encounters of eligible patients. This served to remind the physicians to refer the right patients (English or Spanish-speaking patients between 2- and 6 years) at the right time (during the encounter). The alert also prompts the physicians to indicate if the patient would like to be contacted about PriCARE. This generates an automatic EMR message to the PriCARE coordinator with the patient's contact information thereby eliminating the additional requirement for the physician to send a message. Figure 1 shows the alert. Extensive literature demonstrates the efficacy of EMR alerts in improving processes of care (Fathima et al., 2014; Reis et al., 2017; Sahota et al., 2011; Shojania et al., 2021).

#### Strategy 2 (Direct Advertising to Patients)

The second strategy was a patient-focused strategy in which we prepared patients to be active participants in the PriCARE referral process by direct patient advertising. A Cochrane review of 20 programs found that such mass media efforts effectively influence healthcare utilization (Grilli et al., 2002). Thus, we sent a one-time MyChart message advertising PriCARE to all 2–6-year-old English and Spanish-speaking patients who had signed up for MyChart. MyChart is a secure website associated with the EMR that provides the patient access to portions of their medical record (test results, medications, etc.) and serves as a platform for communication with the healthcare team. In this clinic, 80% of patients have activated MyChart. We also posted PriCARE flyers in the waiting room and in patient rooms.

#### Strategy 3 (Incentives/Public Recognition)

We identified an absence of incentives or recognition for completing PriCARE referrals as a potential barrier to successful referrals. Learning theories suggest that particular behaviors will be repeated if rewarded with incentives (Grol et al., 2007). These theories support the use of reinforcing strategies such as public reporting and recognition, rewards, and incentives to bring about change. Thus, the third strategy selected was public recognition. Physicians at the clinic are divided into five teams of about 10 physicians each. For this strategy, we launched a monthly team competition where the team with the highest percentage of PriCARE referrals at eligible well-child visits would receive $5 Starbucks gift cards for each team member. We shared team progress with physicians through the weekly clinic announcement emails. We also posted graphs demonstrating team progress on the clinic announcement board and highlighted them during the daily provider huddle at the beginning of each clinic session. This strategy was intended to increase physician referrals through incentives as well as in-clinic competition. In addition, this strategy may increase referrals through the provision of regular data and feedback regarding team performance; mechanistically, this is similar to audit and feedback (Strategy 5) described below.

#### Strategy 4 (Patient Narratives)

**Figure 2.**
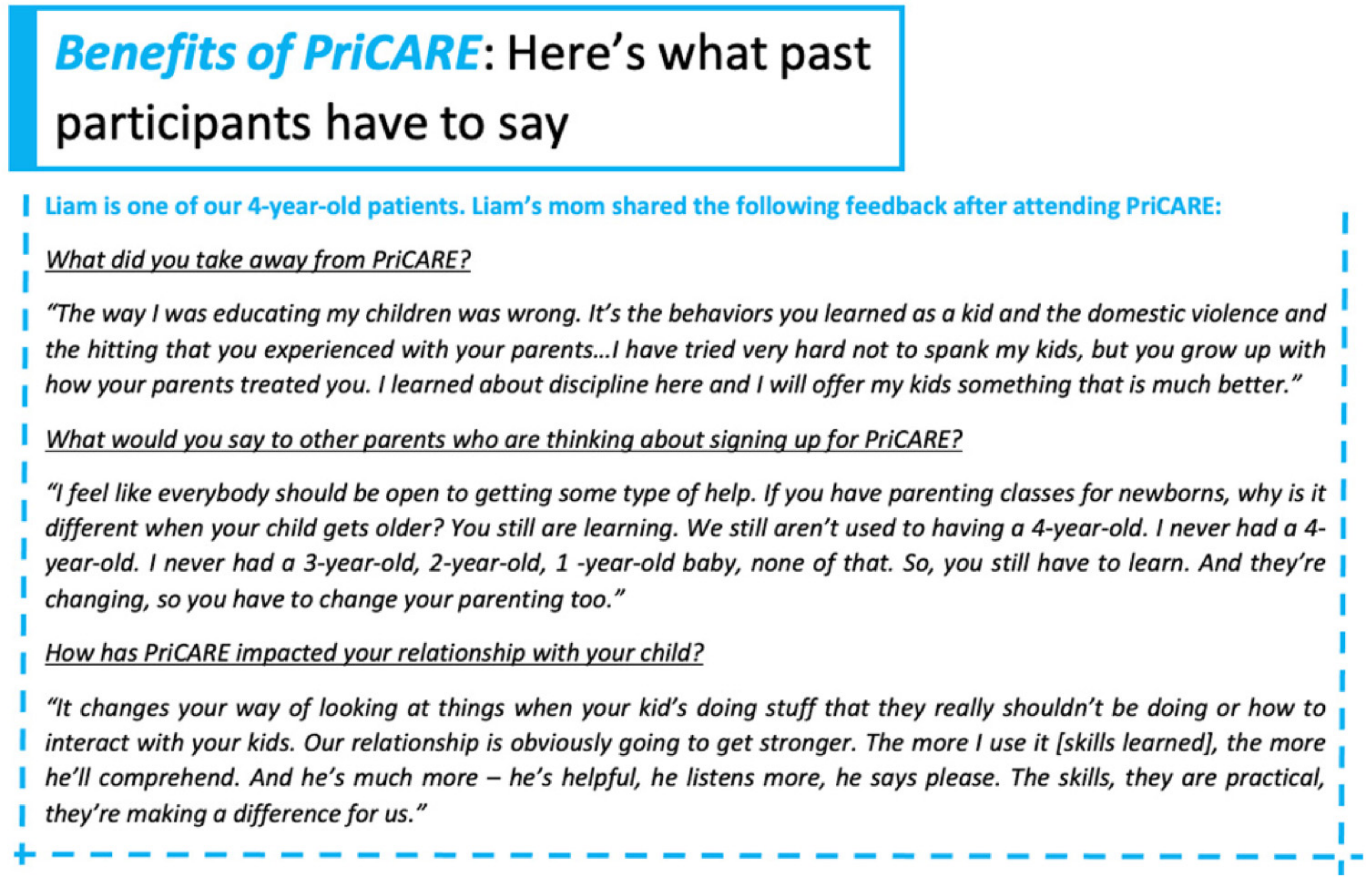
Sample patient narrative included in weekly announcements and posted on clinic announcement board.

During stakeholder interviews, physicians described the many competing priorities during well-child visits and that referring patients to PriCARE may not be a relative priority among all of the expected services that should be delivered during well-child visits. We addressed this barrier by obtaining and disseminating patient testimonial narratives. We obtained patient feedback by interviewing several clinic families who had participated in PriCARE and created testimonial narratives about their experiences with PriCARE which we shared with providers in weekly clinic announcements emails. We also posted the testimonial narratives on the clinic announcement board, as shown in Figure 2. Our hope was that hearing about PriCARE from their patient's perspective may convince physicians that taking time to mention PriCARE during patient encounters is a worthwhile investment. Narratives are easier to comprehend and more engaging than traditional logical-scientific communication (Green, 2006; Kreuter et al., 2007; Stewart & Chambless, 2009). Although we solicited both positive and negative feedback from parents, only the positive comments were included in the testimonials. The majority of the feedback focused on the benefits of PriCARE; the primary negative feedback related to requesting fewer text messages (although most parents said they liked this program feature). To address this suggestion for improvement, we now obtain parent permission before including them in automated-text messages associated with PriCARE.

#### Strategy 5 (Audit and Feedback)

The fifth strategy was an audit of referral rate performance and individual feedback. Multiple behavioral change theories provide guidance on the use of feedback to improve performance (Colquhoun et al., 2017). For instance, communication theories emphasize attention to the individual characteristics of the recipient and motivation theories identify comparative influences as important (Grol et al., 2007; Wensing & Grol, 2020). Together, these theories suggest that giving individualized comparative feedback that shows how an individual's behavior compares to that of their peers may be effective. Studies show that feedback can effectively modify physician behavior (Hardie et al., 2003; Ivers et al., 2012; Jamtvedt et al., 1996; Robertson & Jochelson, 2006). For this strategy, individual emails were sent to each physician summarizing their individual referral rate from initiation of the EMR alert, as well as how the individual compared to the overall clinic referral rate. In keeping with Brehaut et al.’s (2016) suggested best practices for designing and delivering effective feedback interventions, each physician received multiple instances of feedback to encourage a feedback loop wherein the recipient received initial feedback, had an opportunity to initiate a practice change, and then see the effect of the change. In addition, the feedback was individualized, provided alongside comparators that reinforced the desired behavior change, and supported by an easy to interpret visual display (Brehaut et al., 2016).

### Synergistic Ecological Framework

**Figure 3.**
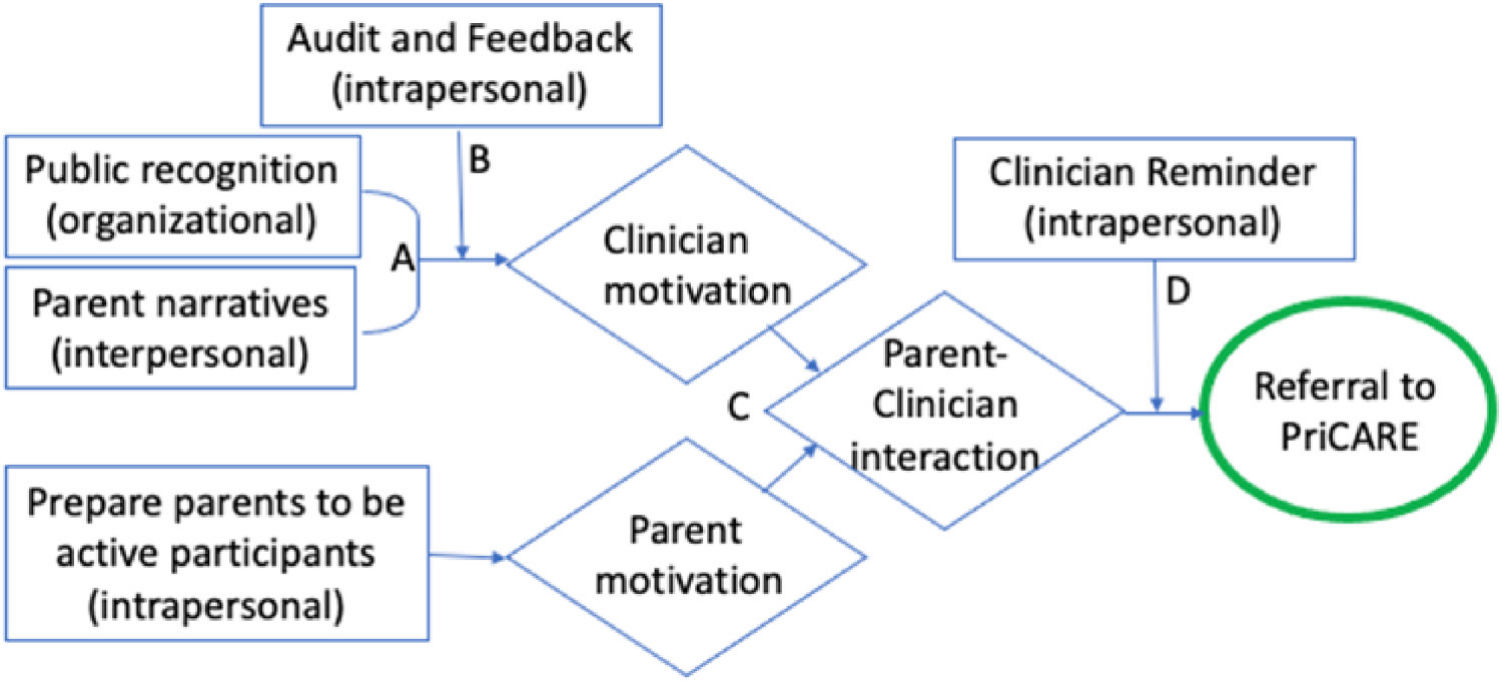
Synergistic relationships among selected discrete implementation strategies to facilitate referrals to Child–Adult Relationship Enhancement in Primary Care (PriCARE). Synergistic relationships among five discrete implementation strategies. Boxes indicate intervention and level of influence (intrapersonal, interpersonal, organizational, community, policy). Diamonds indicate mediators. The oval indicates the outcome. (A) Accumulation, interventions at different levels produce a cumulative impact on a common mediating pathway or set of mediating pathways. (B) Amplification, one intervention increases the target audience's receptivity to other interventions. (C) Convergence, interventions at different levels mutually reinforce each other by altering patterns of interaction among two or more target audiences. (D) Facilitation, one intervention removes the barriers or facilitates the effect of other interventions.

Weiner et al. (2012) propose five strategies for combining interventions in the context of the social-ecological framework to create multifaceted implementation strategies that are synergistic. Figure 3 shows the ways in which the selected discrete strategies described above interact synergistically at multiple levels to facilitate referrals to PriCARE. Organizational incentives/public recognition (Strategy 3) and using patient narratives (Strategy 4) make discrete contributions to motivate physicians, the mediating variable in achieving referrals to PriCARE (*Accumulation Strategy*). Feedback (Strategy 5) augments the impact of these strategies on physician motivation by providing credible information showing discrepancies between the desired and the actual behavior (*Amplification Strategy*). By adding a patient-directed intervention (direct advertising to patients, Strategy 2), the resulting patient motivation and physician motivation reinforce each other and promote physician-patient interactions that result in referrals to PriCARE (*Convergence Strategy*). Finally, reminders (Strategy 1) serve to translate the motivating effect of the former strategies into action (*Facilitation Strategy*).

## Evaluation

The evaluation period spanned 28 weeks and was divided into the pre-implementation period from April 20, 2020 to July 17, 2020 (13 weeks) and the implementation period from July 20, 2020 to October 30, 2020 (15 weeks). The primary targeted outcome was PriCARE referrals. A PriCARE referral was defined as the physician mentioning the PriCARE program at an eligible patient encounter (2–6-year well-child visit with English or Spanish-speaking parents) and inviting the parent–child dyad to participate in PriCARE (i.e., referral), regardless of whether the parent agreed to participate. Referrals were tracked via chart abstraction from the EMR. The 2–6-year well-child visit note template includes the following statement: “The PriCARE/*Cariño* parenting program WAS/WAS NOT discussed with the family.” Physicians must select WAS or WAS NOT. All eligible encounters during the evaluation period were reviewed and selection of “WAS” discussed counted as a referral.

Pre-implementation and implementation mean and median referral rates were calculated. The number of referrals documented in the EMR was the numerator and the total number of eligible encounters was the denominator during each specified period. Chi-square statistics were calculated to test for significance between pre-implementation and implementation referral rates. Referral rates were also calculated for individual physicians before and during implementation. Physicians were only included in the analysis if they had at least one eligible visit during the data collection period.

## Strategy Implementation

**Figure 4.**
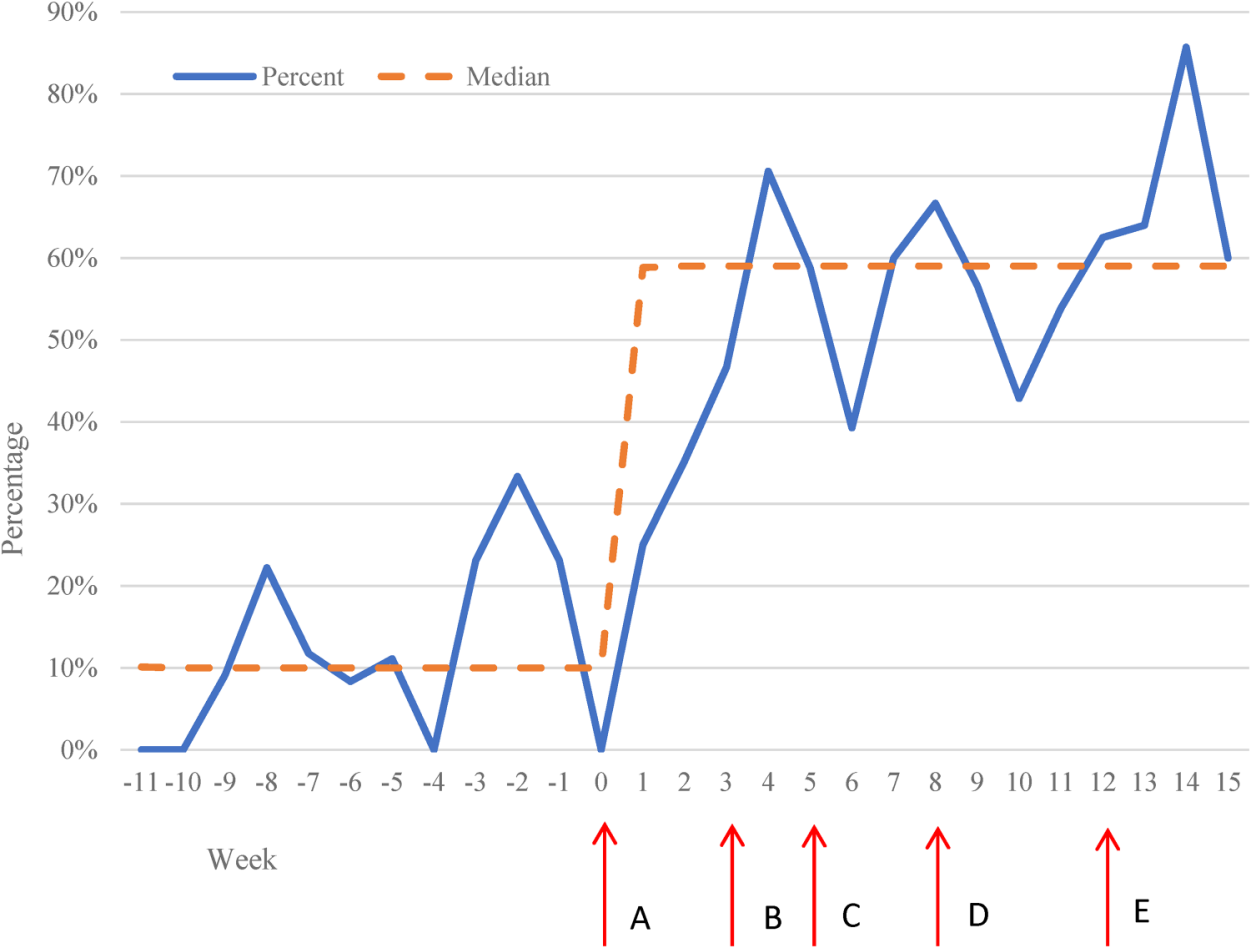
Percentage of Child–Adult Relationship Enhancement in Primary Care (PriCARE) referrals overtime. Dashed line represents the median. Solid line represents the percentage of eligible children who were referred to PriCARE/Cariño. Pre-implementation period (Week 11 to Week 0): median, 10%. Implementation period (Week 1 to Week 15): median, 59%. Implementation Strategies: (A) electronic medical record (EMR) alert (Week 0). (B) MyChart message/flyers (Week 3). (C) Team competition (Week 5). (D) Patient narratives (Week 8). (E) Individual feedback (Week 12).

The five strategies were implemented over a 15-week period and are as follows (Figure 4): At week 0, the EMR alert was activated and during that week, physicians were oriented to the alert during a weekly clinic conference and via email for those not in attendance. At week 3, a one-time MyChart message was sent to all patients in the clinic who were 2–6 years old and identified English or Spanish as their preferred language in the EMR. In addition, program flyers were posted in the waiting room and placed in wall pamphlet holders in the patient rooms for display and distribution. Week 5 marked the beginning of the first of 2 monthlong team competitions; the second competition commenced at week 10. Patient narratives were emailed to physicians in the weekly clinic announcements and posted in the physician workroom every other week starting at week 8 to week 14. Individual feedback emails were sent to physicians during weeks 12–15.

## Results

A total of 131 eligible visits were recorded during the pre-implementation period (April 20, 2020–July 17, 2020) and referrals occurred at 13% (N = 17) of eligible visits. The median referral rate during the pre-implementation period was 10%. During the pre-implementation period, of the 31 physicians included in the analysis, 19% (N = 6) performed equal to or better than the overall clinic mean performance; 80% (N = 25) did not refer to PriCARE at any eligible visit.

During the implementation period (July 20, 2020–October 30, 2020), a total of 315 eligible visits were recorded and the referral rate increased to 55% (N = 172). The median referral rate during the 15-week implementation period was 59% (Figure 4). Difference in referral rate between pre-implementation and implementation was statistically significant (13% vs. 55%, *p* < .001). During the implementation period, of the 47 physicians included in the analysis, a total of 62% (N = 29) performed equal to or better than the overall clinic mean performance, with 23% (N = 11) making referrals to PriCARE during all eligible visits. In contrast, a total of 38% (N = 18) performed worse than the overall clinic, with 13% (N = 6) making no referrals during their eligible visits.

## Considerations for Future Research and Dissemination

This practical implementation report provides a model for the development of multifaceted, multilevel implementation strategies targeting the referral process for the integration of a parenting intervention in primary care. Through implementation of five synergistic strategies, we increased referrals by physicians from 13% to 55%. A steady referral stream is critical to the long-term success of the program, particularly in the absence of funded support for recruitment. While we hope to achieve even higher referral rates, a rate of 55% for all eligible visits is excellent. Such implementation efforts may be even more successful in practices with fewer physicians as it is challenging to influence the behavior of more than 50 physicians, many of whom have clinics only one-half day per week.

In contrast to the “kitchen sink” approach in which multiple strategies are selected from a menu without an overarching conceptual model of how they may operate together, a distinguishing feature of the synergistic approach is the consideration of how the strategies operate at different levels of influence, and, the modeling of how the component strategies work together in complementary ways. The systematic development of multilevel, multifaceted strategies has been noted as a priority to improving implementation and dissemination of evidence-based interventions (Powell et al., 2019), and our report demonstrates a systematic approach driven by theory, evidence, and a synergistic ecological framework reflective of the type of work that is needed. Other communities may select different implementation strategies, but the application of developing and applying this synergistic approach should translate across communities.

In addition to the practical application, there is a clear need for continued development and evaluation of implementation strategies to promote the uptake of evidence-based parenting interventions into general use. A critical component of developing and refining strategies is to unpack and understand the mechanisms through which existing strategies work (Lewis et al., 2018; Powell et al., 2019; Williams, 2016). In this case, it would have been informative to collect quantitative, qualitative, or mixed methods data about how and why these strategies improved the screening and referral process. For example, we could have measured whether referrals were increased by appealing to clinician motivation, influencing parent motivation, or improving the parent–clinician interaction, in addition to exploring other potential mechanisms that were not hypothesized in Figure 3.

## Limitations

There are several limitations to consider when interpreting this practical implementation report. First, additional data would have been helpful in understanding why the five selected strategies did not appear to influence the behavior of the six physicians who made no referrals during eligible encounters in the implementation period. Interviewing this group may have uncovered targets for additional strategies. It is also possible that additional rigorous evaluation may not support the conceptual model depicting the posited complementary interactions of the selected strategies. Perhaps, rather than acting synergistically, one of the strategies could have inadvertently diminished the effect of another strategy. For instance, it is possible that a physician may believe there is no need to mention the program directly during the encounter knowing that all eligible patients received direct advertising about the intervention via MyChart and clinic flyers.

In addition to this need for more evaluation, there are other limitations specific to the execution of our selected strategies. Because we do not know how many patients read the MyChart message, we are unable to estimate the possible impact of this strategy. Based on the number of patients who have activated MyChart, we know that at best, this form of direct advertising to patients only reached 80% of the population. Furthermore, Spanish-speaking families are less likely to use MyChart and therefore they may have been disproportionally less likely to be impacted by this strategy. Another limitation is that because the *Cariño* adaptation was completed around the same time this project started, no Spanish-speaking families had completed the intervention and were therefore not included in the patient testimonials. Future testimonial narratives will include Spanish-speaking participants. We also acknowledge that our decision to not include the negative feedback about PriCARE in the narrative testimonials was not evidence based. While the literature supports that narratives are more compelling than traditional logical-scientific communication (Green, 2006; Kreuter et al., 2007; Stewart & Chambless, 2009), there is no evidence to justify focusing only on positive attributes.

The pandemic context must also be considered in interpreting this report. Pre-implementation and implementation data collection and execution of the strategies all took place during the COVID-19 pandemic (April 20, 2020–October 30, 2020). The PriCARE/*Cariño* groups that had already started in early 2020 were transitioned to a virtual platform in April 2020 and thereafter, PriCARE/*Cariño* was delivered virtually. There were no pauses in recruitment due to the pandemic. However, we recognize the stress of the pandemic may have influenced physician referral behavior, especially early in the pandemic and therefore the improved referral rates may not be entirely attributable to the strategies.

Finally, we recognize that even after implementation of the multilevel strategies, still only 55% of eligible patients were referred. This reflects that there are additional unidentified barriers and facilitators that a more robust approach may have revealed. One contributing factor may have been our failure to include a significant number of frontline clinicians in our stakeholder interviews: only 19% of clinicians responded to our single invitation to provide feedback. In the interest of time and resources, and because the small number of interviews identified multiple opportunities for intervention, we did not solicit additional feedback. Although this method was not comprehensive, the pragmatic approach we present here was successful: the improvement in referral rate was significant and sufficient to support ongoing PriCARE/*Cariño* groups in the clinic.

## Conclusions

The evidence in support of the effectiveness of parenting interventions to improve short- and long-term child health and mental health outcomes is extensive. And yet, the way in which these evidence-based interventions are implemented matters: an evidence-based intervention implemented without an effective screening, referral, and enrollment process will make little impact on improving the lives of children and families on a broad scale. Although delivery in coordination with pediatric primary care is a promising step toward dissemination, there is a pronounced lack of focus on how to do this. Further research related to implementation strategies that support the screening and referral process necessary to support sustained delivery of parenting interventions in primary care is critical to achieving the public health impact these evidence-based interventions have the potential to deliver.
